# Prediction of childhood overweight and obesity at age 10–11: findings from the Studying Lifecourse Obesity PrEdictors and the Born in Bradford cohorts

**DOI:** 10.1038/s41366-023-01356-8

**Published:** 2023-08-04

**Authors:** Nida Ziauddeen, Paul J. Roderick, Gillian Santorelli, Nisreen A. Alwan

**Affiliations:** 1 School of Primary Care, Population Sciences and Medical Education, Faculty of Medicine, University of Southampton, Southampton, UK; 2 NIHR Applied Research Collaboration Wessex, Southampton, UK; 3 Bradford Institute for Health Research, Bradford Royal Infirmary, Bradford, UK; 4 NIHR Southampton Biomedical Research Centre, University of Southampton and University Hospital Southampton NHS Foundation Trust, Southampton, SO16 6YD UK

**Keywords:** Epidemiology, Risk factors

## Abstract

**Background:**

In England, 41% of children aged 10–11 years live with overweight or obesity. Identifying children at risk of developing overweight or obesity may help target early prevention interventions. We aimed to develop and externally validate prediction models of childhood overweight and obesity at age 10–11 years using routinely collected weight and height measurements at age 4–5 years and maternal and early-life health data.

**Methods:**

We used an anonymised linked cohort of maternal pregnancy and birth health records in Hampshire, UK between 2003 and 2008 and child health records. Childhood body mass index (BMI), adjusted for age and sex, at 10–11 years was used to define the outcome of overweight and obesity (BMI ≥ 91st centile) in the models. Logistic regression models and multivariable fractional polynomials were used to select model predictors and to identify transformations of continuous predictors that best predict the outcome. Models were externally validated using data from the Born in Bradford birth cohort. Model performance was assessed using discrimination and calibration.

**Results:**

Childhood BMI was available for 6566 children at 4–5 (14.6% overweight) and 10–11 years (26.1% overweight) with 10.8% overweight at both timepoints. The area under the curve (AUC) was 0.82 at development and 0.83 on external validation for the model only incorporating two predictors: BMI at 4–5 years and child sex. AUC increased to 0.84 on development and 0.85 on external validation on additionally incorporating maternal predictors in early pregnancy (BMI, smoking, age, educational attainment, ethnicity, parity, employment status). Models were well calibrated.

**Conclusions:**

This prediction modelling can be applied at 4–5 years to identify the risk for childhood overweight at 10–11 years, with slightly improved prediction with the inclusion of maternal data. These prediction models demonstrate that routinely collected data can be used to target early preventive interventions to reduce the prevalence of childhood obesity.

## Introduction

Overweight and obesity during childhood is associated with adverse health consequences in adulthood including diabetes, hypertension and coronary heart disease [1]. The rate of obesity in children has increased tenfold worldwide between 1975 and 2016 with 50 million girls and 74 million boys aged 5 to 19 years estimated to be living with obesity in 2016 [2]. The prevalence of overweight and obesity (defined using the UK 1990 population monitoring cut points for overweight (≥ 85th centile) and obesity (≥ 95th centile)) [3] measured as part of the National Child Measurement Programme (NCMP) had remained relatively stable at around 22.9% in children aged 4–5 years (Reception) since 2006/07 till 2019/20, but increased to 27.7% in 2020/21 before decreasing to 22.2% in the 2021/22 results [4]. The prevalence in children aged 10–11 years (Year 6) has been increasing slowly over time since 2009/10 but the increase was more pronounced in 2020/21 with 40.9% of children classified as having overweight and obesity [5]. Although the prevalence decreased to 37.7% in the 2021/22 data, the prevalence remains higher than that prior to the pandemic in 2019/20 (35.2%) [4].

The prevalence of obesity in children aged 10–11 years was more than double that in children aged 4–5 years. Children living in the most deprived areas (13.3% at Year R and 27.5% at Year 6) in England were more than twice as likely to have obesity than children in the least deprived areas (6.0% and 11.9% respectively). This deprivation gap has shown an increase over time from the 2006/07 to the 2019/20 academic year [6]. These high rates of obesity in children are of concern due to the increased risk of persistence of weight status into adulthood [7, 8].

Analysis of 12,076 children from the Millennium Birth Cohort showed that children with overweight at 5 years had a one-third chance of returning to a healthy weight, one-third chance each of remaining overweight or of developing obesity at 11 years; whereas children with obesity at 5 years had a nearly 70% chance of remaining with obesity at 11 years [9]. According to data from NCMP in 2012/13 in South Gloucestershire, a fifth of healthy weight children (20.5%) at Reception had overweight or obesity (≥ 85th centile) by Year 6, 30.3% of overweight children and 68% of children with obesity at Reception had obesity (≥95th centile) by Year 6. However, 42.7% of children with overweight and 11.7% with obesity were a healthy weight by Year 6 [10]. Findings remained consistent on tracking of NCMP weight status from Reception (age 4–5 years) to Year 6 (age 10–11 years) using data from four local authorities with larger representation from deprived and ethnic minority communities [11]. Analysis of NCMP data in Southampton was consistent with previous analysis showing that the difference in prevalence of overweight and obesity between Year R and 6 are greater in children living in more deprived areas [12].

After weight and height are measured in schools as part of NCMP, parents receive a feedback letter informing them of the child’s weight status which includes resources to encourage healthy eating, physical activity and wellbeing. Although the feedback increased parental awareness and recognition of child overweight, it did not result in notable lifestyle changes [13]. A systematic review of interventions for childhood overweight identified school-based interventions combining diet and physical activity have the greatest effectiveness [14] with the majority of interventions in childhood targeting children 6–12 years. Identifying children at risk of remaining or developing overweight is key to effective intervention and providing targeted support. In previous analysis we developed and externally validated models in children up to 2 years of age to predict overweight at age 4–5 years using maternal and early life predictors [15, 16]. In the present analysis, we aimed to develop and validate prediction models of childhood overweight and obesity at age 10–11 years (Year 6) using weight and height measurements at age 4–5 years (Year R) as well as maternal antenatal and birth data. The data is routinely collected as part of healthcare records including the measurement of weight status by the school nursing service in England.

## Methods

We developed and internally validated the prediction model utilising the SLOPE (Studying Lifecourse Obesity PrEdictors) dataset. SLOPE is a population-based anonymised linked cohort of maternal antenatal and birth records and child health records for births registered at University Hospital Southampton (UHS), in the South of England between 2003 and 2018. UHS provides maternity care to residents in the city of Southampton and the surrounding areas of Hampshire. Child healthcare for the same area is provided by two community National Health Service (NHS) trusts; Solent and Southern Health. Only singleton pregnancies with feasible gestational age, maternal weight and maternal height measurements were included in this analysis. All the variables described below are routinely collected for pregnant women and children receiving healthcare in the study region.

We externally validated the prediction models using data from the Born in Bradford (BiB) cohort. BiB is a longitudinal multi-ethnic birth cohort study which recruited 12,453 women comprising 13,776 pregnancies between 2007 and 2010 in Bradford, in the North of England [17].

### Outcome measurement

**Fig. 1:**
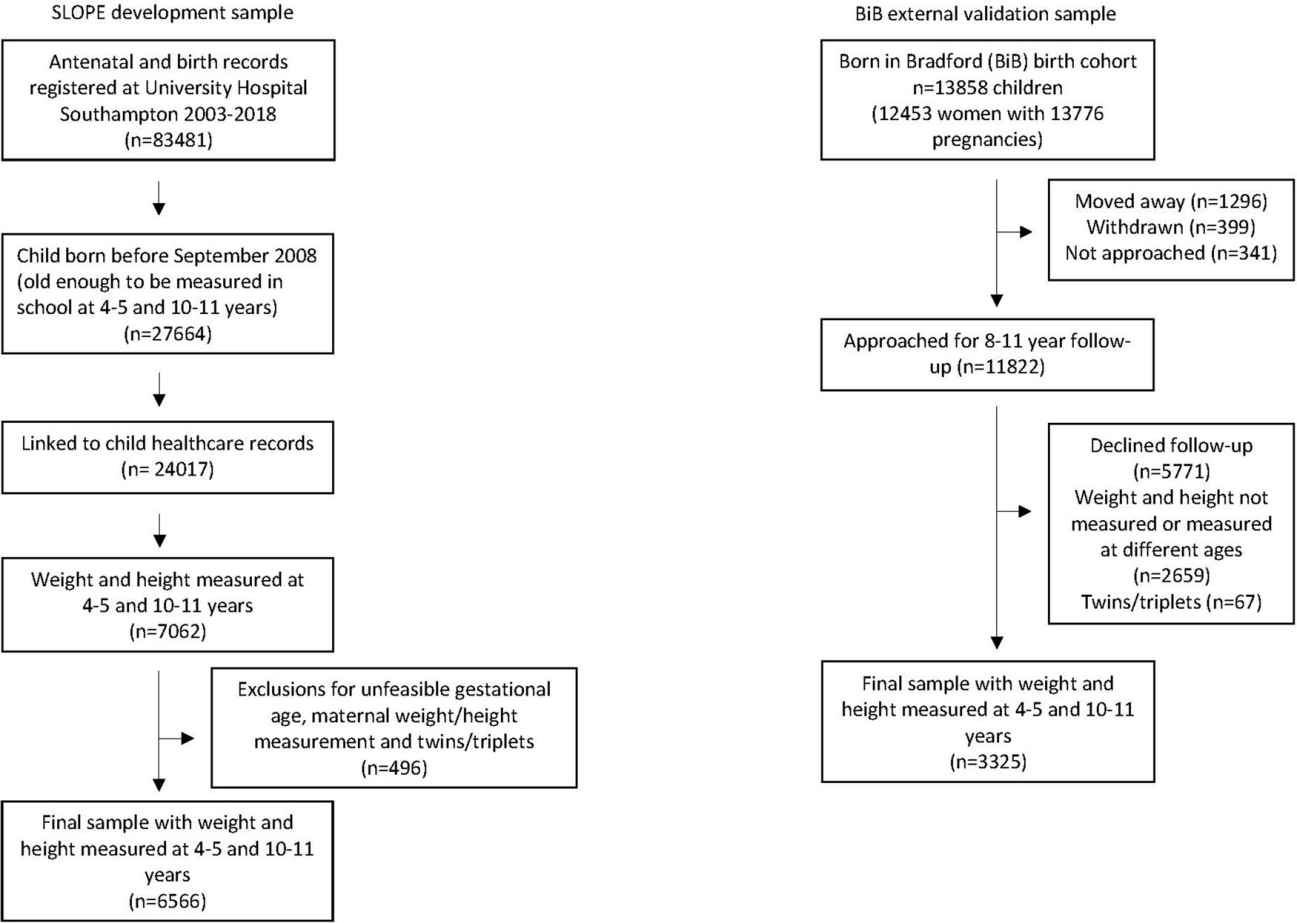
PRISMA flow diagram. Flow diagram showing the eligible sample.

As part of the NCMP, the height and weight of children in all state-maintained schools in England are measured by school nurses at Year R (4–5 years) and Year 6 (10–11 years) [6]. All children with a valid weight and height measurement at both stages (Year R and 6) constituted the sample for outcome (*n* = 6566) (Fig. 1). BMI was calculated as weight/height^2^ and converted to age- and sex- adjusted BMI z-scores according to the UK 1990 growth reference charts [18]. The cut-off of 91st percentile (z-score + 1.33) was used to specify the outcome of childhood overweight and obesity. This cut-off is the most relevant to healthcare professionals in the UK, given it is the one used for national guidance on the clinical management of childhood overweight [3, 19] and used to provide feedback to parents as part of NCMP.

### Candidate predictors

The prediction model was developed in stages, starting with data available at 4-5 years and then adding in data from the first antenatal appointment and birth to check if this improved the prediction.

Year R (age 4–5 years): Weight and height measured in school and gender recorded as part of NCMP measurement by school nurses.

First antenatal (booking) appointment: The first antenatal booking appointment is recommended to ideally take place by the 10th week of gestation, according to NICE Guidelines [20]. Maternal age (in years) was calculated from date of birth in the electronic clinical records (before anonymisation). Maternal BMI was calculated using weight in kilograms measured by the midwife and self-reported height. Smoking was self-reported as current smoker, ex-smoker or non-smoker. Highest maternal educational qualification was categorised as secondary (GCSE) and under, college (A levels) and university degree or above. Self-reported ethnicity was recorded under 16 categories and condensed to White, Mixed, Asian, Black/African/Caribbean and Other. Employment status was categorised as employed, unemployed and in education. Intake of folic acid supplements was categorised as taking before becoming pregnant, started taking once pregnant and not taking supplement. Parity was recorded as the number of previous live births reported and condensed to 0, 1, 2 and ≥ 3 for this analysis. Maternal first language English and partnership status was categorised as yes or no.

Birth: Birthweight (grams) was measured by healthcare professionals at birth using scales that have been calibrated for clinical use. Gestational age was based on a dating ultrasound scan which takes place between 10 weeks and 13 weeks 6 days gestation [20]. Mode of birth was categorised as vaginal and caesarean.

### Statistical analysis

All analysis was performed using Stata v17 [21]. As some women had more than one pregnancy in the dataset, clustering by mother was adjusted for by including a clustering indicator in the model to generate cluster-robust standard errors. The percentage of missing data was low for the antenatal and birth data (< 1% and ethnicity 9%) and thus complete case analysis was performed.

Stepwise backward elimination was used to select variables to be included in the model [22]. This automatic selection procedure starts with the full model (including all candidate predictor variables (Table 1)) and sequentially removes variables based on a series of hypothesis tests. Variables are removed if the *p*-value for a variable exceeds the specified significance level which was set at 0.157 (equivalent to the Akaike information criterion (AIC)) [23] to reduce the risk of overfitting.

**Table 1:** Summary of baseline characters (candidate predictors) and outcome for the SLOPE sample.

		Full sample	< 91st centile	≥ 91st centile
Stage recorded	Variable	Mean ± SD	Mean ± SD	Mean ± SD
	*n*	6566	4849	1717
Booking appointment	Maternal age at booking, years	28.1 ± 5.9	28.1 ± 5.9	27.9 ± 6.1
Booking appointment	Maternal BMI at booking, kg/m^2^	25.2 ± 5.2	24.5 ± 4.7	27.3 ± 6.0
Birth	Birthweight, kg	3.4 ± 0.6	3.4 ± 0.6	3.5 ± 0.6
Birth	Gestation at birth, days	278 ± 14	278 ± 14	278 ± 13
		% (95% CI)	% (95% CI)*	% (95% CI)*
Booking appointment	Maternal smoking status at booking			
	Never smoked	47.5 (46.3–48.7)	49.8 (48.4–51.3)	40.9 (38.6–43.3)
	Ex-smoker	32.5 (31.4–33.7)	32.2 (30.8–33.5)	33.5 (31.3–35.8)
	Current smoker	20.0 (19.0–21.0)	18.0 (16.9–19.1)	25.6 (23.5–27.7)
Booking appointment	Maternal highest educational attainment			
	University degree or above	19.6 (18.7–20.6)	21.8 (20.6–23.0)	13.6 (12.0–15.4)
	College (A levels)	39.9 (38.7–41.4)	39.7 (38.3–41.1)	40.6 (38.2–42.9)
	Secondary school or below	40.5 (39.3–41.7)	38.6 (37.2–39.9)	45.8 (43.4–48.2)
Booking appointment	Maternal employment status at booking			
	Employed	67.5 (66.3–68.6)	68.7 (67.4–70.0)	64.1 (61.8–66.4)
	Unemployed	30.5 (29.4–31.7)	29.6 (29.2–30.9)	33.2 (31.0–35.5)
	Student or in training	2.0 (1.7–2.3)	1.7 (1.4–2.2)	2.6 (1.9–3.5)
Booking appointment	Maternal ethnicity			
	White	90.8 (90.0–91.5)	91.6 (90.8–92.4)	88.4 (86.7–89.9)
	Mixed	1.1 (0.9–1.4)	1.1 (0.8–1.4)	1.3 (0.8–2.0)
	Asian	6.0 (5.4–6.6)	5.6 (4.9–6.2)	0.7 (0.6–0.9)
	Black/African/Caribbean	1.0 (0.8–1.3)	0.7 (0.4–1.0)	1.9 (1.2–2.6)
	Other	1.1 (0.8–1.3)	1.0 (0.7–1.3)	1.2 (0.7–1.9)
Booking appointment	Intake of folic acid supplements			
	Taking prior to pregnancy	29.7 (28.6–30.8)	31.2 (29.9–32.6)	25.5 (23.5–27.7)
	Started taking once pregnant	56.1 (54.9–57.3)	55.6 (54.2–57.0)	57.6 (55.1–59.9)
	Not taking supplement at booking	14.1 (13.3–15.0)	13.1 (12.2–14.1)	16.9 (15.2–18.8)
Booking appointment	Maternal first language English	97.9 (97.6–98.3)	98.1 (97.6–98.4)	97.6 (96.7–98.2)
Booking appointment	Single parent at booking	9.2 (8.6–10.0)	8.7 (7.9–9.5)	10.7 (9.3–12.3)
Booking appointment	Parity at booking			
	0	44.4 (43.2–45.7)	45.4 (44.0–46.8)	41.6 (39.2–44.0)
	1	35.0 (33.9–36.2)	34.8 (33.4–36.2)	35.8 (33.5–38.1)
	2	13.4 (12.6–14.2)	13.4 (12.5–14.4)	13.3 (11.7–15.0)
	≥ 3	7.1 (6.5–7.8)	6.4 (5.7–7.1)	9.4 (8.0–10.9)
Birth	Mode of birth			
	Vaginal	78.1 (77.1–79.1)	78.7 (77.6–79.9)	76.3 (74.2–78.3)
	Caesarean section	21.9 (20.9–22.9)	21.3 (20.1–22.4)	23.7 (21.7–25.8)
Birth	Child sex			
	Male	50.5 (49.3–51.8)	49.3 (47.9–50.7)	54.2 (51.8–56.5)
	Female	49.5 (48.2–50.7)	50.7 (49.3–52.1)	45.8 (43.5–48.2)
4–5 years	Overweight (≥ 91st centile)	14.6 (13.7–15.5)	5.1 (4.5–5.7)	41.5 (39.1–43.8)
10-11 years	Overweight (≥ 91st centile)	26.1 (25.1–27.2)	-	100.0
4–5 and 10–11 years	Childhood overweight (≥ 91st centile) at both stages	10.8 (10.1–11.6)	5.1 (4.5–5.7)	41.5 (39.1–43.8)

^*^95% CI is calculated using the cii proportions command in Stata.

Logistic regression was used to develop the models. Fractional polynomials were used to investigate non-linear relationships between continuous candidate predictors and the outcome. Events per variable (EPV) was used to ensure that the sample size is large enough to avoid issues related to precision and over-fitting. Based on the rule of thumb of 20 EPV, there were sufficient cases of the outcome to develop a prediction model at all the stages [24, 25].

Internal validation: Internal validation was carried out using bootstrapping (1000 repetitions) to provide stable estimates with low bias and an estimate of the expected optimism that can be used to weight down the model parameter estimates [26].

External validation: BiB children aged 7–11 years were followed up using a multi-method approach between 2017 and 2020 (BiB Growing Up Study) [27] as part of which anthropometric measurements were collected by trained researchers. Written informed consent was collected at baseline and follow-up and for continued routine data linkage. Data was available for 3325 children at 10–11 years.

Estimated risk of overweight and obesity at 10-11 years was calculated. There was no missing data in predictor values at Year R and birth but missing data in pregnancy predictor values ranged from 18 to 24%. Missing pregnancy predictor values were imputed using MICE. The percentage of missing data was highest for maternal BMI and this was used to decide the number of imputations (25 imputations with 10 iterations per imputed dataset) [28]. The results from analyses of each of the imputed datasets were combined to produce estimates and confidence intervals that incorporate the uncertainty of imputed values.

Model performance: Model performance was assessed using discrimination and calibration. Discrimination is a measure of how well the model differentiates between individuals [24]. The area under receiver operating characteristic curve (AUC) was used to summarise the overall discriminatory ability of the models. The AUC was classified as: 0.6–0.7 poor, 0.7–0.8 fair, 0.8–0.9 good and 0.9–1.0 excellent. Calibration measures how well the predicted outcome of the model agrees with the observed outcome on average. The predicted probability (x-axis) is plotted against the observed outcome proportion (y-axis) for each tenth of predicted risk (ensuring ten equally sized groups). The slope of a line fitted through the points on the graph is the calibration slope and has been calculated for the models. The calibration slope would be one in a perfectly calibrated model [29].

Prediction models tend to be optimistic in the data used for developing the model due to overfitting. Heuristic shrinkage factors were calculated for each model [30] and the regression coefficients from the models were multiplied by the shrinkage factor to adjust the models for optimism.

Sensitivity, specificity, positive predictive value (PPV) and negative predictive value (NPV) were calculated at multiple risk score cut-off points as no standard criteria for identifying a risk threshold exist for the prediction of childhood obesity.

### Calculating risk score

The log-odds (Y) can be calculated using the regression equation:

$${\mathrm{Y}} = {\mathrm{constant}} + [{\mathrm{estimate}}_1 \times {\mathrm{predictor}}_1] + [{\mathrm{estimate}}_2 \times {\mathrm{predictor}}_2]+ \ldots \ldots \ldots \ldots \ldots \ldots. + [{{\mathrm{estimate}}}_{\mathrm{n}} \times {\mathrm{predictor}}_{\mathrm{n}}]$$

The log-odds (Y) is then converted into probability (P) as follows:

$${{{\mathrm{P}}}} = 1/[1 + {{{\mathrm{exp}}}}( - {{{\mathrm{Y}}}})]$$

where P is the probability of developing the outcome and Y is the log-odds estimated using the model.

### Ethics approval

Ethical approval for SLOPE was granted by the Health Research Authority (HRA) approval (IRAS 242031). Ethical approval for the Born in Bradford cohort was granted by the Bradford Research Ethics Committee (ref. 07/H1302/112 for baseline and 16/YH/0320 (IRAS 207543) for Growing Up).

## Results

**Fig. 2:**
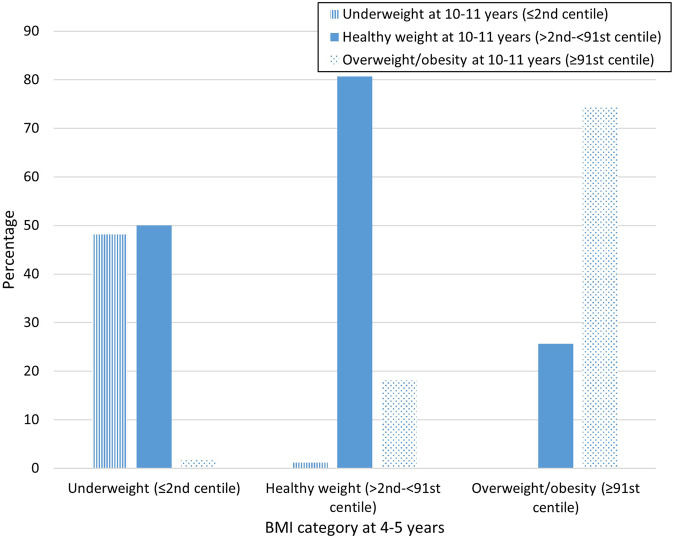
Percentage in each BMI category at 10–11 years by BMI category at 4–5 years. The percentage in each BMI category at Year 6 (10–11 years) by BMI category at Year R (4–5 years).

Of the 6566 children included in the model development, 958 (14.6%) and 1717 (26.1%) had overweight (≥ 91st centile for age and sex) at 4–5 and 10–11 years respectively, with 712 children (10.8%) with overweight at both 4–5 years and 10–11 years. Baseline characteristics are summarised in Table 1. The majority of children in the healthy weight range (> 2nd–< 91st centile) at 4–5 years remained in that range at 10–11 years (80.7%) and similarly for children with overweight and obesity (≥ 91st centile) (74.3%) (Fig. 2).

Mean maternal age at booking was 28.1 years (standard deviation (SD) 5.9). Mean maternal BMI at booking was 25.2 kg/m^2^ (SD 5.2). Over 50% of women reported being ex- (32.5%) or current- (20.0%) smokers. A fifth of the women had a university degree or a higher qualification, and over two–thirds were employed at the first antenatal (booking) appointment. Nine percent of mothers reported being a single parent at the booking appointment.

The prediction models for the risk of childhood overweight and obesity are presented in Table 2. BMI at 4–5 years and child sex were strong predictors of overweight and obesity at 10–11 years and were included in both models. Several pregnancy factors were retained in the model – maternal age, maternal BMI, smoking status, highest educational attainment, employment status and maternal ethnicity, all recorded in the first antenatal appointment. Transformations were identified for child BMI at 4–5 years, maternal age at booking and maternal BMI at booking. No variables recorded at birth (birthweight, gestational age at birth, mode of birth) were retained in the model as the p-values for these variables exceeded the specified significance level.

**Table 2:** Intercept and logistic regression coefficients of the final prediction models for overweight and obesity (≥ 91st centile) in children aged 10–11 years.

	Year R predictors only (*n* = 6566)		Year R + pregnancy predictors (*n* = 5955)	
	Coefficient	Shrunken coefficient	Coefficient	Shrunken coefficient
Constant	−1.18	−0.88	−1.83	−1.69
BMI at 4–5 years	1.00	1.00	0.99	0.97
Child sex				
Male	Ref	Ref	Ref	Ref
Female	−0.29	−0.29	−0.34	−0.33
Maternal age at booking, years			0.02	0.02
Maternal BMI at booking, kg/m^2^			0.07	0.07
Maternal smoking status at booking				
Never smoked			Ref	Ref
Ex-smoker			0.19	0.19
Current smoker			0.51	0.50
Maternal highest educational attainment				
University degree or above			Ref	Ref
College (A levels)			0.40	0.39
Secondary school or below			0.49	0.48
Maternal employment status at booking				
Employed			Ref	Ref
Unemployed			0.06	0.06
Student or in training			0.55	0.54
Maternal ethnicity				
White			Ref	Ref
Mixed			−0.05	−0.05
Asian			0.98	0.96
Black/African/Caribbean			0.74	0.73
Other			0.79	0.77
Parity at booking				
0			Ref	Ref
1			0.05	0.05
2			−0.25	−0.25
≥ 3			−0.02	−0.02
Transformations				
BMI at 4–5 years	BMI - 16.2		BMI - 16.20	
Maternal age at booking			Maternal age - 27.9	
Maternal BMI at booking			Maternal BMI - 25.2	
Model Performance				
AUC on development	0.82 0.80–0.83		0.84 0.83–0.85	
AUC on external validation	0.83 0.82–0.85		0.85 0.84–0.86	
Calibration	1.00 0.94–1.06		1.00 0.94–1.06	
Calibration in the large (CITL)	0.00 −0.07 to 0.07		0.00 −0.07 to 0.07	
Craig and Uhler’s R2	0.364		0.405	
Brier	0.1373		0.1305	

**Fig. 3:**
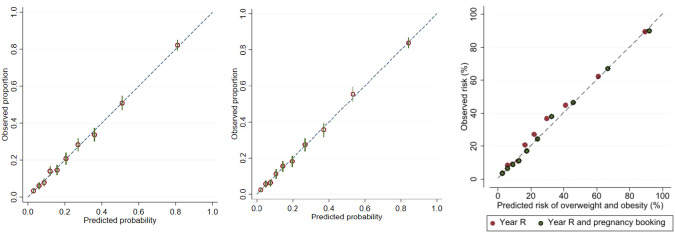
Calibration plots of the prediction models. Calibration plot of the prediction model at 4–5 years (left), including pregnancy factors (middle) and on external validation (right).

Discrimination (AUC) improved from 0.82 with only BMI at 4–5 years and sex as predictors to 0.84 when adding in pregnancy factors (Table 2). The calibration across all models were consistently strong as evidenced by the calibration slope and the gradient (Fig. 3). The estimated shrinkage factors was 0.98 for all models suggesting that only a small percentage of the model fit was noise. The shrunken coefficients and intercepts are presented in Table 2.

AUC on external validation was comparable to that on development for both models. Both models were well-calibrated but agreement was slightly better for the model incorporating pregnancy factors (Fig. 3).

The percentage of children identified as at risk of childhood overweight, sensitivity, specificity, PPV and NPV for different risk score cut-offs are presented in Table 3. As there is no agreed cut-off in the literature on what constitutes ‘high risk’ of future childhood overweight or obesity, a 30% risk threshold was used as deemed the most appropriate by the parameters reported. For example, using a 30% risk cut-off in the Year R model identifies 31.3% of children as at risk, with a sensitivity of 65.8%, specificity of 80.9%, PPV of 55.0% and NPV of 87.0%. When using the model with pregnancy factors, a 30% risk cut-off identified 37.7% at risk, with a sensitivity of 71.3%, specificity of 74.2%, PPV of 49.5% and NPV of 87.9%. The inclusion of pregnancy factors leads to higher sensitivity but not specificity. Specificity and PPV is improved at higher thresholds for both models but with reduced sensitivity. Parameters in the pregnancy model at a cut-off of 40% was similar to that for the Year R model at a 30% cut-off.

**Table 3:** Predictive parameters for the outcome of overweight and obesity (≥ 91st centile) in children aged 10–11 years.

Cut-point	% at or above cut-point	Sensitivity	Specificity	Positive predictive value (PPV)	Negative predictive value (NPV)
Year R (4–5 years)					
≥ 15.0	57.0	86.3 84.5–87.8	53.4 52.0–54.8	39.6 38.0–41.2	91.6 90.6–92.6
≥ 20.0	46.0	80.1 78.2–82.0	66.1 64.7–67.4	45.5 43.7–47.3	90.4 89.4–91.3
≥ 25.0	37.6	72.6 70.4–74.7	74.8 73.5–76.0	50.4 48.5–52.4	88.5 87.5–89.5
≥ 30.0	31.3	65.8 63.5–68.1	80.9 79.8–82.0	55.0 52.8–57.2	87.0 86.0–88.0
≥ 35.0	25.6	58.7 56.3–61.0	86.1 85.1–87.1	60.0 57.6–62.4	85.5 84.5–86.5
≥ 40.0	21.5	53.1 50.7–55.4	89.7 88.8–90.5	64.5 62.0–67.0	84.4 83.3–85.3
Year R (adding in pregnancy factors)					
≥ 15.0	58.0	88.1 86.4–89.6	52.7 51.3–54.1	39.7 38.2–41.3	92.6 91.5–93.5
≥ 20.0	49.3	82.4 80.5–84.2	62.4 61.1–63.8	43.7 42.0–45.5	90.9 89.9–91.9
≥ 25.0	43.0	77.2 75.2–79.2	69.1 67.8–70.4	47.0 45.1–48.8	89.6 88.5–90.5
≥ 30.0	37.7	71.3 69.1–73.4	74.2 72.9–75.4	49.5 47.5–51.4	87.9 86.9–88.9
≥ 35.0	33.3	66.2 63.9–68.5	78.4 77.2–79.5	52.0 49.9–54.1	86.8 85.7–87.7
≥ 40.0	29.6	61.3 58.9–63.6	81.2 80.1–82.3	53.6 51.3–55.8	85.5 84.5–86.6

## Discussion

We have developed and both internally and externally validated prediction models at 10–11 years using data routinely collected in England at 4–5 years. We then incorporated routinely collected data from earlier time-points starting from early pregnancy. Our analysis shows that it is possible to predict childhood overweight and obesity at age 10–11 (Year 6) at age 4–5 (Year R) with good discrimination (AUC 0.82 on development and 0.83 on external validation). The inclusion of routinely available maternal pregnancy data improves the model discrimination (AUC 0.84 on development and 0.85 on external validation). Both models were well calibrated on development and external validation.

As part of the NCMP, parents are provided feedback on their child’s weight status after the measurements at both Year R and 6; but proactive follow-up only occurs in children at extremes of the weight distribution (centiles < 0.4 or ≥ 99.6) [31]. Recommended proactive follow-up involves offering personalised advice, follow-up measurement and services to support healthier weight. However, proactive follow-up in underweight or overweight children not falling into the extreme centiles is dependent on the local authorities and varies across the country [32].

The proportion of parents taking recommended action after feedback about their child’s weight status remains low which may be linked to parental recognition of weight status [13]. Despite increase in parental recognition of childhood overweight and obesity on receiving feedback, recognition still remained low at 38% [13]. Due to different population monitoring and clinical assessment cut-offs, parents of children with BMI in the 85th–< 91st percentile are being informed that their child is of healthy weight. In our dataset, 494 children were in the 85th–< 91st centiles at Year R and 44% of these children were over the 91st centile by Year 6. Socioeconomic circumstances is likely to play an important role in how much resource is available to parents towards achieving or maintaining a healthy weight, particularly with rising child poverty and food insecurity in England [33]. Prediction and risk stratification may help direct resources like healthy eating family vouchers or other financial or social support interventions towards those at highest risk.

Although the model incorporating pregnancy data had better discrimination (AUC 0.84) than the models using data available at Year R alone (AUC 0.82), both models had good discrimination and calibration. A built-in prediction algorithm in the routine child health system once the Year R measurement is recorded could help identify those most at risk of maintaining an overweight status or switching to one by Year 6. The use of routine data in the development of these prediction equations means that these can be readily implemented. Although it may be more straightforward to use the Year R only model due to current recording practices, there is value in linking maternal pregnancy records with child health records as routine practice. This would help to evolve care to incorporate consideration of the increasing evidence on longer term health impacts to both mother and child during the preconception, pregnancy and early life periods.

A systematic review has previously identified eight models for the prediction of risk of overweight and obesity in children, four of which predicted risk in children aged 6-13 years [34]. Gender and maternal BMI were risk factors in three out of the four prediction models but no predictor was common across all four models. None of the models were developed in the UK and the only model that could be applied to routinely collected data in the UK had poor discrimination (AUC 0.64). A recent prediction model developed using cohort data in Scotland to predict obesity at 12 years included maternal BMI, indoor smoking, equivalised income quintile, child sex, child BMI at 5–6 years and adverse childhood experiences [35]. Maternal BMI, child sex and child BMI (at 4–5 years in our analysis and 5–6 years in the Scottish model) are established risk factors and included in both the Scottish and our model. The other predictors included in the Scottish model were not considered for inclusion in our model as it not routinely collected in healthcare data and thus was not available in our dataset. Similarly, prediction models developed using cohort data in the Netherlands [36] and Australia [37] include predictors that are not routinely collected in the UK and thus cannot be easily applied to practice.

A key strength of this analysis is the development of prediction models in a large population-based sample and external validation in a population with different characteristics which enhances the generalisability. SLOPE is a relatively large population-based cohort of women from all socioeconomic and ethnic backgrounds resident in Southampton and surrounding areas of Hampshire and thus representative of the local population. Southampton is more deprived than average [38] but about half of the women included in this analysis reside in surrounding areas of Hampshire which is less deprived. Bradford and Southampton are both relatively deprived cities [38] and the use of data from more deprived areas is a strength given the higher prevalence of overweight and obesity in more deprived areas [6]. The BiB cohort is not representative of the UK but is representative of the local population and has similarities with other UK cities with high levels of ethnic minority groups. We used robust statistical methods to develop the models (retained continuous variables as continuous, investigated variable transformations using multivariable fractional polynomials and corrected for optimism by calculating model shrinkage) and to assess the performance of the models including external validation.

There was a low percentage of missing data in the variables considered for inclusion in the prediction models at the antenatal and birth stage (< 1% and ethnicity 9%). However, early life variables could not be considered for inclusion due to the high percentage of missingness (99% for breastfeeding, 65% for early life weight at 1 or 2 years). Outcome data was not available for a high proportion of children who were old enough to be measured in school. Factors contributing to this potentially include changes in recording practices; a child had moved and was no longer under the care of the community trust; were not attending state school or the child NHS number (required for linkage) was not recorded with the measurement. However, the prevalence of overweight and obesity was similar to the national prevalence (~22% at Year R and ~33% at Year 6 using the 85th percentile cut-off).

A definitive method for identifying a risk threshold could not be identified from the literature, and so we were guided by the sensitivity, specificity, PPV and NPV as well as the number of individuals identified as high risk based on this threshold. For example, for outcome at Year R, the specificity and sensitivity are comparable at a risk threshold of 25% but this identifies around 38% of the sample at risk whereas the outcome prevalence is 26.1%. A risk threshold of 30% using Year R would identify around 31.3% of the sample at risk with higher specificity but lower sensitivity, increase in PPV and slight decrease in NPV. Comparable specificity, sensitivity, PPV and NPV is achieved at higher risk thresholds (35% or 40%) when adding in pregnancy data.

The PPV for our Year R only model at the 30% risk threshold is 55% and the NPV is 87% meaning that a large proportion of children identified at risk will not develop overweight or obesity. The relatively high NPV provides confidence that very few children identified as low risk will develop overweight or obesity. As resources are limited, targeted behavioural, environmental, social or financial support interventions to prevent obesity are needed. There is already a deprivation gradient in childhood obesity [5, 12], therefore using such prediction tools particularly in deprived areas where there is high prevalence of child poverty and food insecurity could direct resources to supporting the families most in need. Targeted prevention interventions are unlikely to produce harms provided we examine the population impact and cost effectiveness of using a risk estimation tool based on routinely collected data as a decision strategy.

## Conclusion

These prediction models can be applied at 4–5 years to identify the risk for later childhood overweight at 10–11 years. The inclusion of maternal pregnancy data slightly improves the prediction. These models demonstrate that utilising routinely collected healthcare data can form the basis of a risk identification system to strengthen the long-term preventive element of early years care by quantifying future obesity risk in families.

### Supplementary information

Supplementary Table 1

## Data Availability

The ethical approval for SLOPE from the University of Southampton and the Health Research Authority restricts public sharing of the raw data used in this study. To request access conditional on approval from the appropriate institutional ethics, research governance processes and data owners, please email rgoinfo@soton.ac.uk. Data requests can be made directly to Born in Bradford by completing an expression of interest form available from https://borninbradford.nhs.uk/research/how-to-access-data/ and submitting it to the BiB Programme Director (rosie.mceachan@bthft.nhs.uk).
