# Supplementary material for: Prediction of childhood overweight and obesity at age 10–11: findings from the Studying Lifecourse Obesity PrEdictors and the Born in Bradford cohorts
Source: Int J Obes (Lond). 2023 Aug 4;47(11):1065–73. doi: 10.1038/s41366-023-01356-8 (PMC10599986; doi:10.1038/s41366-023-01356-8)
Supplement: Supplementary file 1 — Supplementary Table 1 [file 41366_2023_1356_MOESM1_ESM.docx]

Supplementary Table 1: Intercept and regression coefficients of the prediction models for overweight and obesity (≥91st centile) in children aged 10-11 years

|  | Year R predictors | | Year R + pregnancy + birth predictors | |
| --- | --- | --- | --- | --- |
|  | Coef | 95% CI | Coef | 95% CI |
| n | 6566 |  | 5955 |  |
| Constant | -1.18 | -1.28 to -1.09 | -1.83 | -2.04 to -1.61 |
| BMI at 4-5 years | 1.00 | 0.94 to 1.07 | 0.99 | 0.92 to 1.05 |
| Child sex |  |  |  |  |
| Male | Ref |  | Ref |  |
| Female | -0.29 | -0.42 to -0.16 | -0.34 | -0.48 to -0.20 |
| Maternal age at booking, years |  |  | 0.02 | 0.01 to 0.03 |
| Maternal BMI at booking, kg/m^2^ |  |  | 0.07 | 0.06 to 0.09 |
| Maternal smoking status at booking |  |  |  |  |
| Never smoked |  |  | Ref |  |
| Ex-smoker |  |  | 0.19 | 0.02 to 0.35 |
| Current smoker |  |  | 0.51 | 0.31 to 0.72 |
| Maternal highest educational attainment |  |  |  |  |
| University degree or above |  |  | Ref |  |
| College (A levels) |  |  | 0.40 | 0.18 to 0.62 |
| Secondary school or below |  |  | 0.49 | 0.27 to 0.72 |
| Maternal employment status at booking |  |  |  |  |
| Employed |  |  | Ref |  |
| Unemployed |  |  | 0.06 | -0.11 to 0.24 |
| Student or in training |  |  | 0.55 | 0.09 to 1.01 |
| Maternal ethnicity |  |  |  |  |
| White |  |  | Ref |  |
| Mixed |  |  | -0.05 | -0.72 to 0.62 |
| Asian |  |  | 0.98 | 0.68 to 1.28 |
| Black/African/Caribbean |  |  | 0.74 | -0.04 to 1.52 |
| Other |  |  | 0.79 | 0.08 to 1.51 |
| Parity at booking |  |  |  |  |
| 0 |  |  | Ref |  |
| 1 |  |  | 0.05 | -0.12 to 0.22 |
| 2 |  |  | -0.25 | -0.49 to -0.02 |
| ≥3 |  |  | -0.02 | -0.34 to 0.30 |
|  |  | |  | |
| Transformations |  | |  | |
| BMI at 4-5 years | BMI - 16.2 | | BMI - 16.2 | |
| Maternal age at booking |  | | Maternal age - 27.9 | |
| Maternal BMI at booking |  | | Maternal BMI - 25.2 | |
